# Psychological screening, providing social security or strengthening training? How can government and labor unions protect the mental health of young employees

**DOI:** 10.3389/fpubh.2023.1261286

**Published:** 2023-12-04

**Authors:** Yuntao Bai, Lan Wang, Yanzhe Bi

**Affiliations:** ^1^Business School, Shandong Management University, Jinan, China; ^2^Center of Emergency Management, Chongqing Academy of Governance, Chongqing, China; ^3^Health School, Shandong University of Traditional Chinese Medicine, Jinan, China

**Keywords:** mental health, differential game, government, labor unions, social benefits

## Abstract

As the mental health problems of young employees become more and more prominent, the government and labor unions need to take measures to protect the mental health of young employees. Considering that the main mental health safeguard measures are divided into psychological screening, providing social security and strengthening training, this article constructs a differential game model under these three modes. The balanced efforts and social benefits of the government and labor unions under the three modes are obtained, and the applicable conditions of various mental health protection modes are compared. The results show that if the government pays a lower cost, the government gets the highest economic benefit under the training mode, followed by the security mode, and the government gets the lowest economic benefit under the psychological screening mode. If the reputation of the labor unions improved by its efforts is low, the equilibrium benefits of the labor unions under psychological screening and providing security are the same, and greater than the equilibrium benefits under the intensive training mode. If the labor unions have a higher reputation enhanced by its efforts, the balance return of the labor unions under the guarantee mode is the highest, followed by the balance return under the intensive training mode, and the balance return of the labor unions under the psychological screening mode is the lowest.

## 1 Introduction

The rate of mental health problems among young employees has increased. Many factors may contribute to mental health problems in young employees, such as job stress, job imbalance, career development pressure, financial pressure, and job-related uncertainty (1). In addition, the extensive use of modern technology and social media may also have an impact on the mental health of young employees. The exact percentages vary by region and study. According to the World Health Organization, anxiety affects more than 200 million people worldwide each year, and young people are among the hardest hit (2). The high rates of mental health issues among young employees are also a reminder to take mental health issues more seriously in work and educational settings, and provide the necessary support and resources to help them cope and manage their mental health.

The mental health problems of young employees are caused by many aspects. First, young employees may face the challenge of work pressure. Long hours of intense work can lead to anxiety, depression and physical health problems. Second, in the modern economy, young employees may face career uncertainty. This uncertainty can lead to feelings of anxiety, helplessness and loss. Third, it is a challenge for young employees to balance work and personal life. Fourth, young employees may lack support and recognition in the work environment. This can have a negative impact on their self-esteem and mental health. Fifth, some young employees may face inadequate skills matching. This can lead to anxiety and self-doubt. Only when the causes of psychological problems of young employees are found can targeted interventions be carried out (3). The poor mental health of young employees may lead to a series of consequences, such as decreased job performance, stunted career development, physical health, relationship problems, and overall decreased quality of life.

In order to protect the mental health of young employees, a variety of measures are needed. For example, first, the government and labor unions can conduct regular psychological screening of employees to help identify potential psychological distress and diseases (4). But this method can only screen out a certain percentage of young employees with mental health problems. Second, the government and labor unions can provide security for the life of employees, can help young employees better adapt to the work environment, improve job satisfaction and quality of life, and lay a foundation for their future development (5). However, this method is easy to bring employees negative feelings of sabotage. Third, the government and labor unions can provide training for employees. By strengthening employee training, organizations can improve the skill level and performance of employees, stimulate the potential and creativity of employees, and promote the innovation and development of enterprises. However, training can take up employees’ time and lead to employee dissatisfaction.

Understanding the causes of mental health problems among young employees can not only help young employees better understand the problem, but also provide them with appropriate interventions and support. In the long run, this understanding can help promote the mental health and overall well-being of young employees. Some scholars have studied the causes of mental health problems. These causes mainly include three aspects: external macro environment, corporate environment, and personal conditions. In terms of external macro environment, inappropriate working conditions and challenging socio-economic conditions are the main causes of mental health problems among young female employees in India (6). Public health crises can lead to psychological problems among nurses (7). In terms of corporate environment, the impact of human resource management on employees’ psychological contract (8). How emotional labor affects employees’ mental health (9). The impact of challenging pressure and task performance on the mental health of university employees (10). The negative impact of customer abuse on service employees (11). In terms of personal conditions, gender, age, and type of education lead to anxiety among Chinese teachers during the COVID-19 pandemic (12).

In the face of factors affecting the mental health of young employees, effective solutions must be found. Some scholars have studied how to protect the mental health of employees. These studies mainly focus on treatment methods, leadership types, psychological support, working environment, training, external media, etc. Some scholars have studied how to treat the mental problems of employees. For example, from an ethical and moral perspective, prevention can be more effective, while from the perspective of efficacy and efficiency, treatment can be more effective (13). In terms of leadership types, Roberge and Boudrias (14) studied how empowered leadership active performance regulates the mental stress of employees. Ahmed et al. (15) studied how inclusive leadership protects the mental health of employees during trauma and crisis. In terms of psychological support, Fukuti et al. (16) studied how to protect the mental health of medical workers through psychological support during COVID-19. Li et al. (17) believed that it is necessary to enhance the professional commitment of nursing students to protect their mental health. In terms of working environment, Aboujaoude (18) believed that the mental health of employees can be protected by protecting their privacy. Plugge (19) analyzed how to protect the mental health of prison workers during COVID-19. Torquati et al. (20) studied how to protect the mental health of shift workers. Zhu et al. (21) believed that a high degree of workplace inclusion could buffer the potential negative psychological impact of disability on an individual level. In terms of training, Slobodianiuk (22) believed that psychological training for future teachers should be strengthened. In terms of external media, Zhang et al. (23) believed that social media communication had a positive impact on employees’ psychological state.

However, the mental health status of young employees is constantly changing. Every young employee may face different challenges and pressures, so the psychological issues will be different. With career development and increased work experience, young employees may face higher levels of challenges and responsibilities. At the same time, government and labor unions measures to protect the mental health of young employees are constantly changing. The study does not show such a change. In order to solve the shortcomings of the above research, differential game is adopted in this article. This is a continuous time game. Many scholars have applied it to logistics mode selection (24), environmental governance (25), supply chain (26), etc. This method can clearly describe the dynamic change process of mental health protection. At the same time, this approach has other advantages of game theory. First, the method can analyze the interaction between the government and young employees, and between labor unions and young employees. Second, it can reveal the benefits of protecting the mental health of young employees, and promote the efforts of the government and labor unions to protect the mental health of young employees. Third, governments and labor unions should be encouraged to develop policies to safeguard the mental health of young employees.

Meanwhile, the above research did not analyze the mental health problems of employees from an economic perspective, and did not consider the reputation of the government and labor unions. In order to make up for the shortcomings of the above research, this article analyzes the impact of the protection of employees’ mental health on the reputation of the government and labor unions from the perspective of utility function.

In the selection of game parties, this article chooses the government and the labor union as the game parties. This is because governments and labor unions have an important role to play in improving the mental health of young employees. The government’s role in improving the mental health of young employees is crucial. By developing policies and regulations, providing mental health education and advocacy, establishing networks of mental health services, supporting research and data collection, and providing insurance and benefit programs, governments can help create a more mentally healthy and supportive work environment and improve the mental health of young employees. Meanwhile, labor unions have an important role to play in improving the mental health of young employees. They can help employees cope with psychological stress by providing support and counseling, advocating for employee rights, strengthening employee support networks, and providing mental health insurance and benefit programs to create a healthier and supportive work environment.

To improve the mental health of young employees, the government and labor unions have three modes.

1. Psychological screening mode. Providing psychological screening for employees is an important initiative that can help predict and identify potential mental health problems and take appropriate interventions early (27). For example, young employees can be screened for mental health through questionnaires, psychological assessment tools, and regular health check-ups. Specifically, psychological behavior data can be used to identify and verify people’s psychological conditions (28). However, due to the reluctance to disclose the privacy and data-related data of young employees, psychological screening cannot completely screen out young employees with psychological problems.
2. Provide social security mode. Providing security for young employees is key to maintaining their quality of work and life. For example, young employees can be provided with reasonable pay and benefits, working hours and vacation policies, health and safety guarantees, and so on. Specifically, governments and labor unions should ensure that young employees receive fair and reasonable remuneration packages that reflect their skills and contributions. In addition, comprehensive welfare plans are provided, including medical insurance, pensions, supplementary pensions, etc., to meet their basic living needs (29). However, providing a higher level of protection for young employees is likely to lead to some young employees’ slack in their work.
3. Strengthen training mode. The spending of the government and labor unions can have an impact on education or training (30). Training young employees is important because they are the new force in the organization and have the potential to become future leaders. Specifically, governments and labor unions can provide young employees with an experienced mentor or coach who can provide hands-on experience and guidance. Through the above methods, organizations can strengthen the training of young employees, stimulate their potential, enhance their capabilities, and train new leaders for the future development of the organization.

There are many factors to consider when protecting mental health (31). Each mode of mental health improvement has its own advantages and disadvantages. It is important for the government and labor unions to choose appropriate ways to protect the mental health of young employees. Only by choosing the right mental health improvement mode can we provide the necessary psychological support and resources for young employees to help them cope with pressure, adjust their mentality, maintain positive mental health, and promote the sustainable development of individuals and organizations (32). The relationship between different mental health protection modes is shown in Appendix Figure 1 (See Appendix 1 for details).

## 2 Methodology

### 2.1 Differential game, hypothesis and variable definition

#### 2.1.1 Differential game

The role of game theory is to help us understand and predict the underlying principles of human behavior and decision making, and to provide a formalized framework for solving these problems. It is important for formulating the best strategy, optimizing resource allocation and understanding strategic interactions (33). Differential game, also known as dynamic game or evolutionary game. It is a branch of game theory that is used to study the changes in the game process over time. Unlike static games, differential games focus on the changes in the strategies and behaviors of the participants in the game, and are analyzed over continuous time periods. In differential games, the participants adjust their strategies according to a certain equation or system. This equation or system is usually established based on the dynamic laws of interaction between the participants. By studying the optimal strategies and behaviors of the participants, the evolutionary laws and stable results of the game can be obtained at continuous equilibrium points in time (34).

The application of differential game can help to understand and develop more effective strategies to ensure the mental health of young employees. Differential game focuses on the dynamic change of participants’ behavior and strategy over time, which makes it easier to understand and predict how various factors affecting the mental health of young employees, such as work pressure, interpersonal relationship and job satisfaction, change over time. The following are some possible advantages of using differential game analysis to ensure the mental health of young employees. First, dynamic analysis. Differential game can help us make dynamic prediction and simulation of the mental health of young employees, and observe and evaluate the impact of various factors on the mental health of young employees on the time axis (35). Second, cause-and-effect relationship revealing. Differential game can reveal the interaction and influence relationship between various factors affecting the mental health of young employees, helping us find out the root cause of the problem and put forward the most effective solution. Third, strategy optimization. Differential game provides a systematic method to optimize the mental health strategy of employees, so that we can maximize the mental health of young employees by adjusting relevant factors. Fourth, prevention mechanism construction. Differential game can better predict the influence of various factors on the mental health of young employees, develop preventive management measures to prevent the occurrence of mental health problems of employees. For example, preventive stress reduction activities, mental health education, etc. Fifth, improve the accuracy of management decisions. Through differential game, we can accurately measure and predict the actual effect of various management strategies on the mental health of young employees, making management decisions more scientific and accurate.

#### 2.1.2 Hypothesis

(1) The protection of the mental health of young employees by the government and labor unions is in line with economic principles.

Government and labor union guarantees for the mental health of young employees can be consistent with a number of economic principles, especially those relating to labor markets and human capital. First, it would make labor markets more efficient. Mental health problems can lead to reduced employee productivity and lower quality of work. By providing protection for the mental health of young employees, governments and labor unions can reduce the loss of employee productivity due to mental health problems and improve the efficiency of the labor market. Second, it conforms to the theory of human capital investment. Mental health is a kind of human capital of individuals, which has an important impact on individual’s work ability and career development. By providing mental health protection, governments and labor unions are actually investing in the human capital of young employees to enhance their future working capacity and income levels. Third, health and social security. The concept of social security in economics emphasizes the need for government and organizational support to mitigate the inevitable risk borne by individuals. Mental health problems can have a negative impact on an individual’s economic and social life. The provision of mental health protection by the government and labor unions can help address the risks faced by young employees in this area and provide them with a safe and stable working environment. Fourth, the long-term economic impact. Young employees are important resources for future economic vitality and social development. By focusing on and safeguarding their mental health, governments and labor unions can promote their development and potential release at work, thereby contributing to long-term economic growth and sustainable development of society.

(2) Labor unions are concerned about their reputation.

The reputation of labor unions as organizations representing the rights and interests of employees is crucial to their influence and credibility. Here are a few reasons why labor unions are concerned about their reputation. First, this can involve member recruitment and retention. The reputation of a labor union can play an important role in attracting new members and retaining existing ones. A union with a good reputation attracts more employees and increases membership loyalty and engagement. Second, it can involve government and employer relations. The reputation of labor unions also affects their relations with governments and employers. If labor unions have a good reputation, governments and employers may be more willing to cooperate and dialogue with them to address the rights and interests of employees (36). Thirdly, it can garner public opinion and public support. The reputation of a labor union also affects the level of public recognition and support. A union with a good reputation will be more likely to command public support and sympathy, and thus have more power to fight for rights and push for change.

(3) The mental health status of young employees is constantly changing.

Young adults are an important stage of life development, experiencing great physical, emotional and cognitive changes, as well as being affected by socio-economic changes (37). Therefore, their mental health may also be diverse and dynamic. When young employees enter the workplace, they may need to adapt to new environments and responsibilities, and face new positions, job requirements and work pressures. This shift can have an impact on their mental health, such as anxiety, stress and self-doubt. Young employees are often in the beginning stages of their career path, and they may face uncertainties and challenges in their career development. Uncertain job prospects and career planning stress can have an impact on their mental health. Young employees may face work-life balance issues. They may need to adjust to long working hours, overtime and job stress, while neglecting other important areas of life such as family, social and personal interests. This imbalance can have a negative impact on their mental health. Understanding this dynamic and providing support and protection are critical to maintaining the mental health of young employees. Stakeholders such as governments and labor unions can help young employees cope with and manage changes in mental health by providing adaptive support, promoting work-life balance, providing training and development opportunities, and promoting a healthy work culture (Table 1).

**Table 1:** The main definition of variables and parameters in this article.

Variables and parameters	Specific meaning
Y = {*S,G,T*}	Three modes for improving mental health (psychological screening, provide social security, strengthen training)
Independent variable	
*F*_*Y*1_(*t*)	The level of government efforts under the mental health improvement mode *Y*
*F*_*Y*2_(*t*)	The level of labor union efforts under the mental health improvement mode *Y*
*x*_*Y*1_(*t*)	The reputation of the government under the mental health enhancement mode *Y*
*x*_*Y*2_(*t*)	The reputation of the labor union under the mental health enhancement mode *Y*
Parameter	
*ρ*	The discount rate that occurs over time, the discount factor, 0 ≤ *ρ* ≤ 1
*δ*	The decay rate of reputation, *δ* > 0
*a*_*Y*1_, *a*_*Y*2_	The unit gain to the government or labor union from the improvement of the mental health of young employees. Among them, 1 represents the government and 2 represents the labor union (the same below). *a*_*Y*1_, *a*_*Y*2_ > 0
*l*	The positive impact of the organization’s reputation, *l* > 0
*I_S_*	The difficulty for the government to obtain information on the number of youth units, *I_S_* > 0
*f*_1_, *f*_2_	Employee dissatisfaction caused by unit level psychological screening, *f*_1_, *f*_2_ > 0
*c*_*Y*1_, *c*_*Y*2_	The unit cost of improving the mental health of young employees to governments or labor union, *c*_*Y*1_, *c*_*Y*2_ > 0
*p*_1_, *p*_2_	The probability of government or labor union can screen out troubled a young employees, *p*_1_, *p*_2_ > 0
*β*_1_, *β*_2_	The enhanced reputation for unit effort by government or union, *β*_1_, *β*_2_ > 0
*d*	Resistance of young employees to the unit level of training, *d* > 0
*b*_*T*1_	The contribution of young employees to society by improving the unit level of skill, *b*_*T*1_ > 0
*b*_*G*2_	The passive sabotage caused by the young employees due to accepting unit security, *b*_*G*2_ > 0
Function	
*J_Yi_*(*t*)	The social welfare function of government or labor union under the mental health improvement mode *Y*
*V_Yi_*(*t*)	The social benefits of the government or labor union under the mental health improvement mode *Y*

#### 2.1.3 Variable definition

### 2.2 Differential game of different mental health protection mode

Globalization and the rapid development of information technology have brought increasing pressure to young employees around the world. With the increasingly fierce competition in the world, many young employees around the world are facing great mental pressure for job stability and career development, resulting in an increasing number of young people suffering from mental health problems (2). In response, both governments and labor unions need to take action to support young workers to improve their resilience to stress, which can have a significant impact on their physical and mental health.

The psychological protection of global employees requires unlimited time, mainly because the maintenance of mental health is a lasting process, not a one-time action. It requires sustained attention at all levels, including prevention, intervention and recovery. All three stages take time, so the psychological protection of global employees is unlimited. Moreover, due to the constant change of personal conditions, work environment, work pressure and other factors, it is required that enterprises should always pay attention to the mental health status of employees and provide continuous and personalized psychological protection measures (15). Therefore, psychological protection is a lasting process and should not have a time limit.

The social welfare function of governments and unions consists of the benefits gained from psychological screening, the costs of psychological screening, and the benefits generated by psychological screening. At the same time, this paper mainly selects the reputation of government and labor union as state variables for the following reasons. In differential games, the reputation of government and labor unions is chosen as a state variable because it plays a key role in many social situations. First, reputation is a persistent and stable variable that can be used to describe the performance or behavior of governments and unions over a period of time, and thus predict their likely performance in the future. Second, reputation can influence the decisions of governments and unions. A good reputation may lead to more opportunities, such as gaining more approval ratings. Therefore, understanding reputation can help us understand behavior and decision-making.

In general, the solving steps of differential game include the following stages. First, establish the game model. Identify the participants, define their objective function (or cost function), and the variables (strategies) they can control and influence. Usually, this model is an optimization problem with constraints. Second, according to the optimization theory, the above problem is transformed into the Hamilton-Jacobi-Bellman (HJB) equation, which describes the dynamic behavior of the optimal strategy of the players (38). Third, solve the HJB equation. In many practical cases, the HJB equation is very complex and may not have an explicit solution. At this time, numerical methods (such as finite difference method, finite element method, etc.) are often needed to approximate the solution (39).

#### 2.2.1 Psychological screening

If the government and the labor union can carry out psychological screening of young employees, then the psychological problems of employees can be found in time. And take measures to correct the symptoms of employees, help employees to relieve depression and anxiety, improve the mental health of employees. Young employees work in an enterprise, because of long-term contact, the union has a clearer grasp of the information of the employees. As a result, psychological screening costs are higher for the government than for labor unions. Under the psychological screening mode, the social benefits of the government and the labor union are respectively:


(1) $$J_{S1}=\overset{\infty }{\underset{0}{\int }}\left[a_{S1}F_{S1}\left(t\right)p_{1}-\frac{c_{S1}\ln\left(e+I_{S}\right)}{2}F_{S1}^{2}\left(t\right)+lx_{S1}\left(t\right)\right]\;e^{-\rho t}dt$$

(2) $$J_{S2}=\overset{\infty }{\underset{0}{\int }}\left[a_{S2}F_{S2}\left(t\right)p_{2}-\frac{c_{S2}}{2}F_{S2}^{2}\left(t\right)+lx_{S2}\left(t\right)\right]\;e^{-\rho t}dt$$

Among them, $a_{S1}F_{S1}\left(t\right)p_{1}$ represents the benefits of the government’s psychological screening of young people. $\frac{c_{S1}\ln\left(e+I_{S}\right)}{2}F_{S1}^{2}\left(t\right)$ represents the cost of psychological screening by the government. The government can obtain the information of young employees through questionnaires and interviews. However, it is harder for governments to get information this way than it is for labor unions. Among them, $\ln\left(e+I_{S}\right)$ indicates how difficult it is for the government to get information from young employees. $lx_{S1}\left(t\right)$ represents the social reputation that psychological screening has brought to the government. $a_{S2}F_{S2}\left(t\right)p_{2}$ represents the benefits of the union’s psychological screening of youth groups. $\frac{c_{S2}}{2}F_{S2}^{2}\left(t\right)$ represents the cost of psychological screening by the union. $lx_{S2}\left(t\right)$ represents the social reputation that psychological screening brings to the union. *P*_1_ represents the probability that the government could find a problematic employee. *P*_2_ represents the probability that the union could find a problem employee.

Government and labor union psychological screening of employees can make employees with psychological problems feel that they are taken seriously. Therefore, psychological screening can improve the favorable impression of young employees on the government and labor unions. At the same time, the psychological screening of young employees by the government and labor unions will delay the time of young employees, so this will lead to dissatisfaction of some employees. The change in the reputation of governments and labor unions can be expressed as:


(3) $${\dot{x}}_{S1}\left(t\right)=p_{1}\beta_{1}F_{S1}\left(t\right)-f_{1}F_{S1}\left(t\right)-\delta x_{S1}\left(t\right)$$

(4) $${\dot{x}}_{S2}\left(t\right)=p_{2}\beta_{2}F_{S2}\left(t\right)-f_{2}F_{S2}\left(t\right)-\delta x_{S2}\left(t\right)$$

Among them, $p_{1}\beta_{1}F_{S1}\left(t\right)$ represents psychological screening improves the reputation of the government. $p_{2}\beta_{2}F_{S2}\left(t\right)$ represents psychological screening improves the reputation of labor unions. $f_{1}F_{S1}\left(t\right)$ represents young employees are unhappy with government screening. $f_{2}F_{S2}\left(t\right)$ represents young employees are unhappy with union screenings. $\delta x_{S1}\left(t\right)$ represents a decline in the government’s reputation. $\delta x_{S2}\left(t\right)$ represents a decline in the reputation of labor unions.

#### 2.2.2 Provide social security

If the government and labor unions provide security to employees, it will increase the satisfaction of young employees. But providing protection for workers costs governments and labor unions a lot of money. At the same time, if the employee accepts the guarantee, it will lead to the employee’s passive work. Under the provision of security mode, the social benefits of the government and the labor union are as follows:


(5) $$J_{G1}=\overset{\infty }{\underset{0}{\int }}\left[a_{G1}F_{G1}\left(t\right)-\frac{c_{G1}}{2}F_{G1}^{2}\left(t\right)+lx_{G1}\left(t\right)\right]\;e^{-\rho t}dt$$

(6) $$J_{G2}=\overset{\infty }{\underset{0}{\int }}\left[a_{G2}F_{G2}\left(t\right)-\frac{c_{G2}}{2}F_{G2}^{2}\left(t\right)-b_{G2}F_{G2}\left(t\right)+lx_{G2}\left(t\right)\right]\;e^{-\rho t}dt$$

Among them, $a_{G1}F_{G1}\left(t\right)$ represents the improvement in the mental health of young employees as a result of government protection. $\frac{c_{G1}}{2}F_{G1}^{2}\left(t\right)$ represents the cost of government protection. $lx_{G1}\left(t\right)$ represents the reputation gained by the government under the government-provided security mode. $b_{G2}F_{G2}\left(t\right)$ represents the loss caused by passive work. $a_{G2}F_{G2}\left(t\right)$ represents the improvement in the mental health of young employees as a result of the protection provided by the labor union. $\frac{c_{G2}}{2}F_{G2}^{2}\left(t\right)$ represents the cost of union protection. $lx_{G1}\left(t\right)$ represents the reputation gained by the union under the mode of provide social security.

Many young employees have psychological problems. In such cases, governments and labor unions can enhance their reputations by providing guarantees to employees. The change in the reputation of governments and labor unions can be expressed as:


(7) $${\dot{x}}_{G1}\left(t\right)=\beta_{1}F_{G1}\left(t\right)-\delta x_{G1}\left(t\right)$$

(8) $${\dot{x}}_{G2}\left(t\right)=\beta_{2}F_{G2}\left(t\right)-\delta x_{G2}\left(t\right)$$

Among them, $\beta_{1}F_{G1}\left(t\right)$ represents an increase in the government’s reputation as a result of providing security. $\beta_{2}F_{G2}\left(t\right)$ represents an increase in the reputation of the union as a result of providing security. $\delta x_{G1}\left(t\right)$ represents the decline of the government’s reputation under the mode of providing social security. $\delta x_{G2}\left(t\right)$ represents the decline of the union’s reputation under the mode of providing social security.

#### 2.2.3 Strengthen training

If you strengthen training for employees, then the ability of employees will be improved to a certain extent, so as to obtain a certain psychological satisfaction. At the same time, it costs a certain amount to strengthen training for employees. With the improvement of young employees’ abilities, they can better give back to society. Under the enhanced training mode, the social benefits of the government and the labor union are as follows:


(9) $$J_{T1}=\overset{\infty }{\underset{0}{\int }}\left[a_{T1}F_{T1}\left(t\right)-\frac{c_{T1}}{2}F_{T1}^{2}\left(t\right)+b_{T1}F_{T1}\left(t\right)+lx_{T1}\left(t\right)\right]\;e^{-\rho t}dt$$

(10) $$J_{T2}=\overset{\infty }{\underset{0}{\int }}\left[a_{T2}F_{T2}\left(t\right)-\frac{c_{T2}}{2}F_{T2}^{2}\left(t\right)+lx_{T2}\left(t\right)\right]\;e^{-\rho t}dt$$

Among them, $a_{T1}F_{T1}\left(t\right)$ represents the improvement in the mental health of young employees as a result of the government’s enhanced training for young employees. $\frac{c_{T1}}{2}F_{T1}^{2}\left(t\right)$ represents the cost of increased government training. $b_{T1}F_{T1}\left(t\right)$ represents the contribution of young employees to society through training. $lx_{T1}\left(t\right)$ represents the reputation gained by the government under the mode of enhanced training. $a_{T2}F_{T2}\left(t\right)$ represents the improvement in the mental health of young employees as a result of the union’s enhanced training for young employees. $\frac{c_{T2}}{2}F_{T2}^{2}\left(t\right)$ represents the cost of enhanced training by the union. $lx_{T2}\left(t\right)$ represents the labor union gained a reputation under the mode of enhanced training.

Some young employees have psychological problems because of their limited work ability, it is difficult to get a high-paying position or be recognized by others. In this case, training is needed in order to improve the mental health of employees. However, the training needs to consume a lot of staff time, which causes staff dissatisfaction. Thus, the change in the reputation of the government and labor unions can be expressed as:


(11) $${\dot{x}}_{T1}\left(t\right)=\beta_{1}F_{T1}\left(t\right)-dF_{T1}\left(t\right)-\delta x_{T1}\left(t\right)$$

(12) $${\dot{x}}_{T2}\left(t\right)=\beta_{2}F_{T2}\left(t\right)-dF_{T2}\left(t\right)-\delta x_{T2}\left(t\right)$$

Among them, $dF_{T1}\left(t\right)$ represents the government training took time away from employees and led to employee dissatisfaction. $dF_{T2}\left(t\right)$ represents employee dissatisfaction caused by union training taking up employees’ time. $\beta_{1}F_{T1}\left(t\right)$ represents an increase in the government’s reputation as a result of strengthen training. $\beta_{2}F_{T2}\left(t\right)$ represents an increase in the reputation of the union as a result of strengthen training. $\delta x_{T1}\left(t\right)$ represents the decline of the government’s reputation under the mode of strengthen training. $\delta x_{T2}\left(t\right)$ represents the decline of the union’s reputation under the mode of strengthen training.

## 3 Results

In the differential game, the social welfare of the government and the labor union is not only affected by the control variables and parameters, but also constantly changes with time and the mental health of young employees. In order to better calculate the improvement of the mental health status of young employees and the social benefits of the government and labor unions, the HJB formula is adopted in this article. HJB formula is a partial differential equation, which is the core of optimal control.

### 3.1 HJB formula

The application of HJB equation to the solving process of differential game is usually in the analysis of dynamic strategy problems including time and state. The strategy selection problem of each participant in the game process needs to be formalized into an optimal control problem, in which the control variable is the strategy of each participant. The solving process can be roughly divided into the following steps. First, describe the strategy space. First of all, the strategy space of the participant needs to be described, that is, all possible strategy choices. Second, set the objective function. The goal of the participant in the game process can usually be expressed by an expected utility function. This function depends on the strategy choice of the participant and the strategy choice of other participants. Third, establish the HJB equation. Through the Hamilton minimization principle, the HJB equation describing the system can be obtained. This equation includes the strategy choice of the participant, the strategy choice of other participants and the utility function of the participant (40). Fourth, solve the HJB equation. By solving the HJB equation, the strategy that maximizes the expected utility can be found, which is the optimal strategy of the participant. The dynamic programming method and numerical approximation method in mathematics may be used to solve the HJB equation (41). Finally, by comparing the optimal strategies of different participants, the Nash equilibrium of the differential game can be found, that is, at this equilibrium point, any participant changing his strategy will not improve his utility.

When the psychological screening mode is adopted for mental health protection, the HJB equation under this mode are:


(13) $$\begin{array}{l} \rho V_{S1}=\max_{F_{S1}\left(t\right)}\{\left[a_{S1}F_{S1}\left(t\right)p_{1}-\frac{c_{S1}\ln\left(e+I_{S}\right)}{2}F_{S1}^{2}\left(t\right)+lx_{S1}\left(t\right)\right]\\ \;+\frac{\partial V_{S1}}{\partial x_{S1}}\left[p_{1}\beta_{1}F_{S1}\left(t\right)-f_{1}F_{S1}\left(t\right)-\delta x_{S1}\left(t\right)\right]\} \end{array}$$

(14) $$\begin{array}{l} \rho V_{S2}=\max_{F_{S2}\left(t\right)}\{\left[a_{S2}F_{S2}\left(t\right)p_{2}-\frac{c_{S2}}{2}F_{S2}^{2}\left(t\right)+lx_{S2}\left(t\right)\right]\\ \;+\frac{\partial V_{S2}}{\partial x_{S2}}\left[p_{2}\beta_{2}F_{S2}\left(t\right)-f_{2}F_{S2}\left(t\right)-\delta x_{S2}\left(t\right)\right]\} \end{array}$$

When the mode of providing social security is adopted for mental health protection, the HJB equation under this mode are:


(15) $$\begin{array}{l} \rho V_{G1}=\max_{F_{G1}\left(t\right)}\{\left[a_{G1}F_{G1}\left(t\right)-\frac{c_{G1}}{2}F_{G1}^{2}\left(t\right)+lx_{G1}\left(t\right)\right]\\ \;+\frac{\partial V_{G1}}{\partial x_{G1}}\left[\beta_{1}F_{G1}\left(t\right)-\delta x_{G1}\left(t\right)\right]\} \end{array}$$

(16) $$\begin{array}{l} \rho V_{G2}=\max_{F_{G2}\left(t\right)}\{\left[a_{G2}F_{G2}\left(t\right)-\frac{c_{G2}}{2}F_{G2}^{2}\left(t\right)-b_{G2}F_{G2}\left(t\right)+lx_{G2}\left(t\right)\right]\\ \;+\frac{\partial V_{G2}}{\partial x_{G2}}\left[\beta_{2}F_{G2}\left(t\right)-\delta x_{G2}\left(t\right)\right]\} \end{array}$$

When the strengthen training mode is adopted for mental health protection, the HJB equation under this mode are:


(17) $$\begin{array}{l} \rho V_{T1}=\max_{F_{T1}\left(t\right)}\{\left[a_{T1}F_{T1}\left(t\right)-\frac{c_{T1}}{2}F_{T1}^{2}\left(t\right)+b_{T1}F_{T1}\left(t\right)+lx_{T1}\left(t\right)\right]\\ \;+\frac{\partial V_{T1}}{\partial x_{T1}}\left[\beta_{1}F_{T1}\left(t\right)-dF_{T1}\left(t\right)-\delta x_{T1}\left(t\right)\right]\} \end{array}$$

(18) $$\begin{array}{l} \rho V_{T2}=\max_{F_{T2}\left(t\right)}\{\left[a_{T2}F_{T2}\left(t\right)-\frac{c_{T2}}{2}F_{T2}^{2}\left(t\right)+lx_{T2}\left(t\right)\right]\\ \;+\frac{\partial V_{T2}}{\partial x_{T2}}\left[\beta_{2}F_{T2}\left(t\right)-dF_{T2}\left(t\right)-\delta x_{T2}\left(t\right)\right]\} \end{array}$$

### 3.2 Result of equilibrium

Proposition 1: In the psychological screening mode, the effort degree of government and labor unions to improve the mental health of young employees, and the social benefits of government and labor unions are, respectively (See Appendix 2 for details):


(19) $$F_{S1}^{*}\left(t\right)=\frac{a_{S1}p_{1}+\frac{l}{\rho +\delta }\left(p_{1}\beta_{1}-f_{1}\right)}{c_{S1}\ln\left(e+I_{S}\right)}$$

(20) $$F_{S2}^{*}\left(t\right)=\frac{a_{S2}p_{2}+\frac{l}{\rho +\delta }\left(p_{2}\beta_{2}-f_{2}\right)}{c_{S2}}$$

(21) $$\begin{array}{c} \begin{array}{l} V_{S1}^{*}=\frac{l}{\rho +\delta }x_{S1}+\frac{1}{\rho }a_{S1}\frac{a_{S1}p_{1}+\frac{l}{\rho +\delta }\left(p_{1}\beta_{1}-f_{1}\right)}{c_{S1}\ln\;\left(e+I_{S}\right)}p_{1}\\ \;-\frac{1}{\rho }\frac{c_{S1}\ln\left(e+I_{S}\right)}{2}{\left[\frac{a_{S1}p_{1}+\frac{l}{\rho +\delta }\left(p_{1}\beta_{1}-f_{1}\right)}{c_{S1}\ln\;\left(e+I_{S}\right)}\right]}^{2} \end{array}\\ \;+\frac{1}{\rho }\frac{l}{\rho +\delta }\left(p_{1}\beta_{1}-f_{1}\right)\frac{a_{S1}p_{1}+\frac{l}{\rho +\delta }\left(p_{1}\beta_{1}-f_{1}\right)}{c_{S1}\ln\;\left(e+I_{S}\right)} \end{array}$$

(22) $$\begin{array}{c} \begin{array}{l} V_{S2}^{*}=\frac{l}{\rho +\delta }x_{S2}+\frac{1}{\rho }a_{S2}\frac{a_{S2}p_{2}+\frac{l}{\rho +\delta }\left(p_{2}\beta_{2}-f_{2}\right)}{c_{S2}}p_{2}\\ \;-\frac{1}{\rho }\frac{c_{S2}}{2}{\left[\frac{a_{S2}p_{2}+\frac{l}{\rho +\delta }\left(p_{2}\beta_{2}-f_{2}\right)}{c_{S2}}\right]}^{2} \end{array}\\ \;+\frac{1}{\rho }\frac{l}{\rho +\delta }\left(p_{2}\beta_{2}-f_{2}\right)\frac{a_{S2}p_{2}+\frac{l}{\rho +\delta }\left(p_{2}\beta_{2}-f_{2}\right)}{c_{S2}} \end{array}$$

Conclusion 1: In the psychological screening mode, the balanced efforts of the government and the labor union are proportional to the probability of screening the problematic young employees. At the same time, it is inversely proportional to the level of employee dissatisfaction caused by psychological screening.

Proposition 2: In the mode of providing social security, the effort degree of government and labor unions to improve the mental health of young employees, and the social benefits of government and labor unions are, respectively (See Appendix 3 for details):


(23) $$F_{G1}^{*}\left(t\right)=\frac{a_{G1}+\frac{l}{\rho +\delta }\beta_{1}}{c_{G1}}$$

(24) $$F_{G2}^{*}\left(t\right)=\frac{\left(a_{G2}-b_{G2}\right)+\frac{l}{\rho +\delta }\beta_{2}}{c_{G2}}$$

(25) $$\begin{array}{l} V_{G1}^{*}=\frac{l}{\rho +\delta }x_{G1}+\frac{1}{\rho }a_{G1}\frac{a_{G1}+\frac{l}{\rho +\delta }\beta_{1}}{c_{G1}}-\frac{1}{\rho }\frac{c_{G1}}{2}{\left[\frac{a_{G1}+\frac{l}{\rho +\delta }\beta_{1}}{c_{G1}}\right]}^{2}\\ \;+\frac{1}{\rho }\frac{l}{\rho +\delta }\beta_{1}\frac{a_{G1}+\frac{l}{\rho +\delta }\beta_{1}}{c_{G1}} \end{array}$$

(26) $$\begin{array}{c} \begin{array}{l} V_{G2}^{*}=\frac{l}{\rho +\delta }x_{G2}+\frac{1}{\rho }a_{G2}\frac{\left(a_{G2}-b_{G2}\right)+\frac{1}{\rho +\delta }\beta_{2}}{c_{G2}}\\ \;-\frac{c_{G2}}{2}\frac{1}{\rho }{\left[\frac{\left(a_{G2}-b_{G2}\right)+\frac{l}{\rho +\delta }\beta_{2}}{c_{G2}}\right]}^{2} \end{array}\\ \begin{array}{l} -\frac{1}{\rho }b_{G2}\frac{\left(a_{G2}-b_{G2}\right)+\frac{1}{\rho +\delta }\beta_{2}}{c_{G2}}\\ +\frac{1}{\rho }\frac{l}{\rho +\delta }\beta_{2}\frac{\left(a_{G2}-b_{G2}\right)+\frac{1}{\rho +\delta }\beta_{2}}{c_{G2}} \end{array} \end{array}$$

Conclusion 2: Under the mode of providing social security, the balance effort of the government is inversely proportional to the contribution of the young employees to the society by improving the unit skills.

Proposition 3: In the strengthen training mode, the effort degree of government and labor unions to improve the mental health of young employees, and the social benefits of government and labor unions are, respectively (See Appendix 4 for details):


(27) $$F_{T1}^{*}\left(t\right)=\frac{\left(a_{T1}+b_{T1}\right)+\frac{l}{\rho +\delta }\left(\beta_{1}-d\right)}{c_{T1}}$$

(28) $$F_{T2}^{*}\left(t\right)=\frac{a_{T2}+\frac{l}{\rho +\delta }\left(\beta_{2}-d\right)}{c_{T2}}$$

(29) $$\begin{array}{c} \begin{array}{l} V_{T1}^{*}=\frac{l}{\rho +\delta }x_{T1}+\frac{1}{\rho }a_{T1}\frac{\left(a_{T1}-b_{T1}\right)+\frac{l}{\rho +\delta }\left(\beta_{1}-d\right)}{c_{T1}}\\ \;-\frac{1}{\rho }\frac{c_{T1}}{2}{\left[\frac{\left(a_{T1}-b_{T1}\right)+\frac{l}{\rho +\delta }\left(\beta_{1}-d\right)}{c_{T1}}\right]}^{2} \end{array}\\ \begin{array}{l} \;+\frac{1}{\rho }b_{T1}\frac{\left(a_{T1}-b_{T1}\right)+\frac{1}{\rho +\delta }\left(\beta_{1}-d\right)}{c_{T1}}\\ \;+\frac{1}{\rho }\frac{l}{\rho +\delta }\left(\beta_{1}-d\right)\frac{\left(a_{T1}-b_{T1}\right)+\frac{1}{\rho +\delta }\left(\beta_{1}-d\right)}{c_{T1}} \end{array} \end{array}$$

(30) $$\begin{array}{l} \begin{array}{l} V_{T2}^{*}=\frac{l}{\rho +\delta }x_{T2}+\frac{1}{\rho }a_{T2}\frac{a_{T2}+\frac{l}{\rho +\delta }\left(\beta_{2}-d\right)}{c_{T2}}\\ \;-\frac{1}{\rho }\frac{c_{T2}}{2}{\left[\frac{a_{T2}+\frac{l}{\rho +\delta }\left(\beta_{2}-d\right)}{c_{T2}}\right]}^{2} \end{array}\\ \;+\frac{l}{\rho +\delta }\left(\beta_{2}-d\right)\frac{a_{T2}+\frac{l}{\rho +\delta }\left(\beta_{2}-d\right)}{c_{T2}} \end{array}$$

Conclusion 3: In the strengthen training mode, the balanced efforts of government and labor union are inversely proportional to the resistance of young employees to training.

### 3.3 Numerical analysis

In order to more clearly depict the social benefits of the government and the labor union, this article carries out numerical analysis. Therefore, the following assumptions are made about the values of parameters in this article. The discount factor *ρ* is 0.9. Reputation decay rate *δ* = 0.1. The government gains *a*_*Y*1_ from the improvement of young employees’ mental health is 1.5. Gains *a*_*Y*2_ to the labor union from improved mental health of young employees is 2. The positive impact *l* of unit reputation is 1. Employee dissatisfaction *f*_1_ caused by unit level psychological screening is 0.6. Employee dissatisfaction *f*_2_ caused by unit level psychological screening is 0.4. The probability *p*_1_ that the government can screen out problematic young employees is 0.4. The probability *p*_2_ that the union can detect the problem young employees is 0.5. The difficulty *I_S_* for the government to obtain information on the number of youth units is 2. The resistance *d* of young employees to training at the unit level is 2. The contribution *b*_*T*1_ of young employees to society by improving the unit skill is 3. Negative work sabotage *b*_*G*2_ caused by young employees’ acceptance of unit security is 1.5. Both the government and the union have a reputation *x* of 1.

When the enhanced reputation *β*_1_ for unit effort by government is 1, this article can calculate:


(31) $$V_{S1}^{*}=1+0.054\times \frac{1}{c_{S1}}$$

(32) $$V_{G1}^{*}=1+3.47\times \frac{1}{c_{G1}}$$

(33) $$V_{T1}^{*}=1+6.87\times \frac{1}{c_{T1}}$$

**Figure 1:**
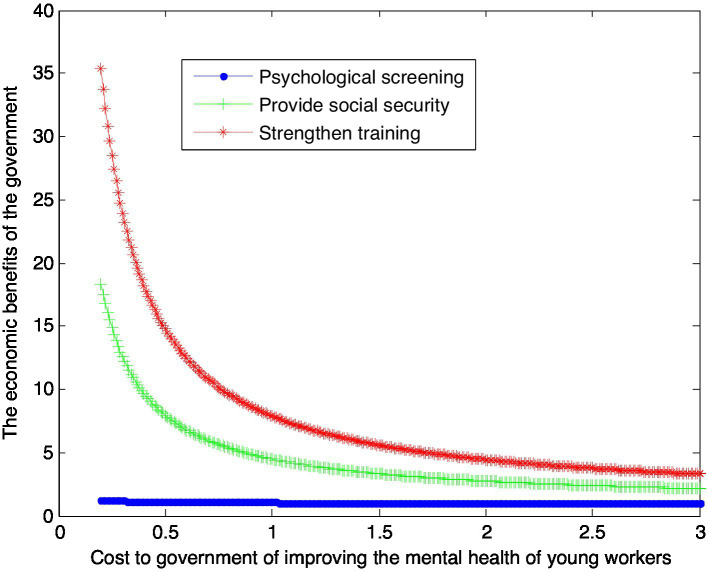
Impact of mental health care costs on government benefits.

This article can make the following graph, named Figure 1.

When the enhanced reputation *β*_1_ for unit effort by government is 2, this article can calculate:


(34) $$V_{S1}^{*}=1+0.224\times \frac{1}{c_{S1}}$$

(35) $$V_{G1}^{*}=1+2.92\times \frac{1}{c_{G1}}$$

(36) $$V_{T1}^{*}=1+10.75\times \frac{1}{c_{T1}}$$

**Figure 2:**
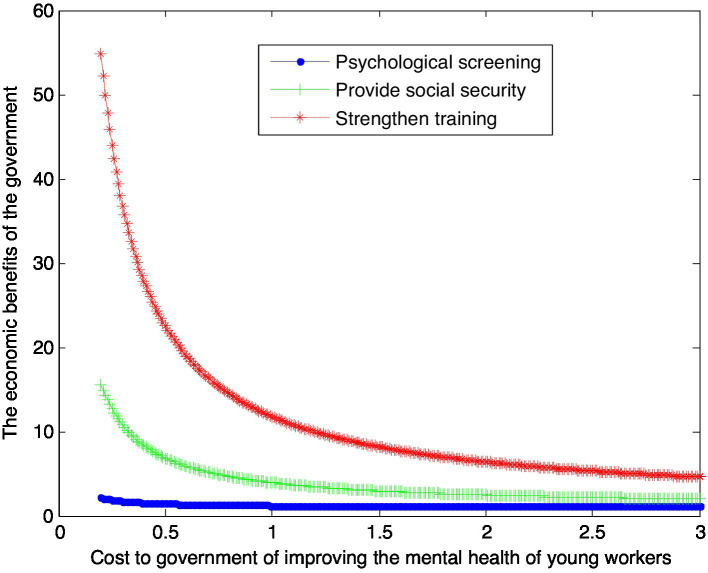
Impact of mental health care costs on government benefits.

This article can make the following graph, named Figure 2.

According to Figures 1, 2, conclusion 4 can be drawn.

Conclusion 4: If the government pays a lower cost, the government gets the highest economic benefit under the training mode, followed by the security mode, and the government gets the lowest economic benefit under the psychological screening mode. However, with the increase of the cost paid by the government, the economic benefits of the government under the three modes gradually decrease and tend to be the same.

When the enhanced reputation *β*_2_ for unit effort by labor union is 1, this article can calculate:


(37) $$V_{S2}^{*}=1+1.25\times \frac{1}{c_{S2}}$$

(38) $$V_{G2}^{*}=1+1.25\times \frac{1}{c_{G2}}$$

(39) $$V_{T2}^{*}=1+0.66\times \frac{1}{c_{T2}}$$

**Figure 3:**
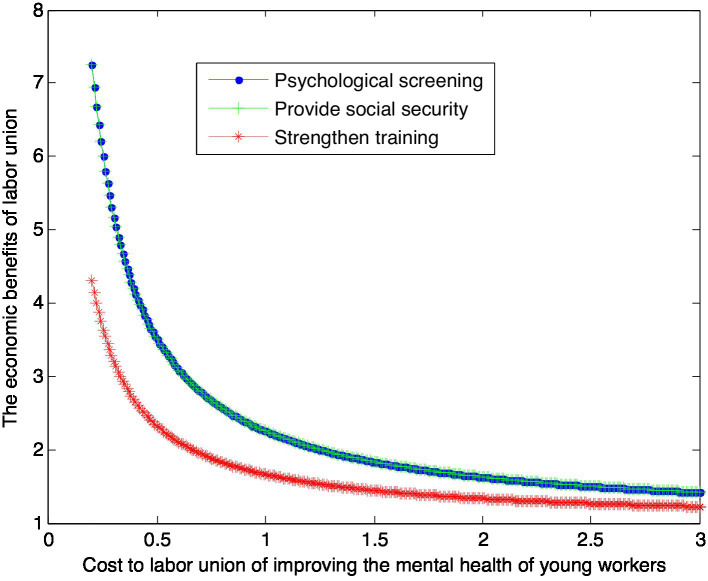
Impact of mental health care costs on labor union benefits.

This article can make the following graph, named Figure 3.

According to Figure 3, conclusion 5 can be drawn.

Conclusion 5: If the reputation of the union improved by its efforts is low, the equilibrium benefits of the union under the psychological screening and providing security modes are the same, and greater than the equilibrium benefits under the intensive training mode. However, with the increase of the cost paid by the government, the equilibrium benefits of the union under the three modes gradually decrease and tend to be the same.

When the enhanced reputation *β*_2_ for unit effort by labor union is 2, this article can calculate:


(40) $$V_{S2}^{*}=1+3.21\times \frac{1}{c_{S2}}$$

(41) $$V_{G2}^{*}=1+4.09\times \frac{1}{c_{G2}}$$

(42) $$V_{T2}^{*}=1+3.33\times \frac{1}{c_{T2}}$$

**Figure 4:**
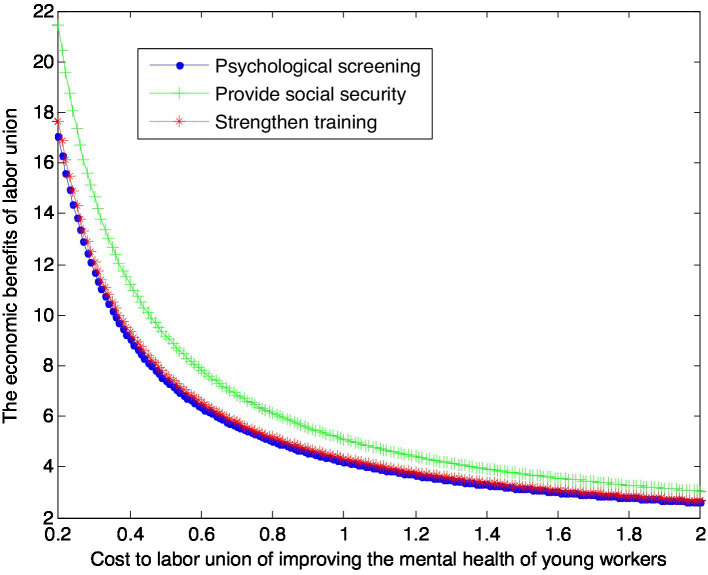
Impact of mental health care costs on labor union benefits.

This article can make the following graph, named Figure 4.

According to Figure 4, conclusion 6 can be drawn.

Conclusion 6: If the labor union has a higher reputation enhanced by its efforts, the labor union under the guarantee mode has the highest equilibrium return, followed by the enhanced training mode, and the labor union under the psychological screening mode has the lowest equilibrium return. However, with the increase of the cost paid by the government, the equilibrium benefits of the union under the three modes gradually decrease and tend to be the same.

## 4 Discussion

According to Conclusion 1, it is possible that the level of government and union effort is proportional to the probability of screening out problematic young employees. There are some parallels with the research of Poddar and Madupalli (42). They believe that companies should actively pay attention to the impact of problematic customers on employee turnover and serve these employees. Conclusion 1 is mainly caused by the following reasons. First, the government and labor unions are willing to invest more money in mental health resources, including mental health education, training and psychological counseling services. This will increase the coverage and quality of screening and improve the probability of finding problematic young employees. Second, the government and labor unions actively promote and raise public awareness of the importance of mental health, encourage young employees to pay attention to their mental health, and encourage them to participate in screening. This will greatly increase the number of people involved in screening and improve the probability of finding problematic employees. Third, governments and labor unions work with mental health agencies and professionals to provide screening services and support, including the provision of psychological assessment tools and support from counselors and therapists. This will improve the accuracy and professionalism of screening and increase the probability of finding problematic employees.

The balanced effort of the government to improve the mental health of young employees is inversely proportional to the contribution of young employees to the society by improving their work skills. Conclusion 2 is different from Ozaki et al. (43). Ozaki et al. (43) believe that workplace psychological distress is related to a sense of social contribution, and workplace mental health promotion should consider employees’ sense of contribution to society. Conclusion 2 is mainly caused by the following reasons. The increased contribution of young employees to society can positively affect the mental health of employees. First, enhance the self-confidence of young employees. By constantly upgrading their skills, young employees can perform better at work and achieve better results. This will increase their confidence and satisfaction and help improve their mental health. Second, reduce the work pressure of young employees. With stronger skills and abilities, young employees are better equipped to deal with challenges and pressures at work. They are able to complete tasks more easily and reduce the negative impact of work stress on mental health. Third, provide a career path. Skills upgrading provides more career development opportunities for young employees. This opportunity for professional development can bring satisfaction and a sense of accomplishment, improving the mental health of employees.

The level of balanced efforts of the government and labor unions is inversely proportional to the level of resistance of young employees to training. This is the main point of Conclusion 3. This is similar to Clercq et al. (44). They argue that the mitigating effect of goal alignment is more pronounced among employees with a lower propensity to learn. Conclusion 3 is mainly caused by the following reasons. The possibility of task variation reduces employees’ motivation to invest in task-specific skills (45). To this end, governments and labor unions should not focus so much on training that they ignore the objections of young employees. In order to reduce the resistance of young employees to training, the following needs to be done. First, improve the content and quality of training. If the training content does not match the actual needs and interests of employees, or the quality of training is not high, it may lead to the resistance of young employees to the training. Second, pay attention to time and cost input. Training may require additional time and resource investment and may interfere with the work and personal lives of young employees, causing them to resist their willingness to participate in training. Third, timely communication and participation. If the government and the labor unions fail to communicate and consult adequately, and if they fail to obtain the views and participation of the employees, the young employees may hold an opposing position to the training. Governments and labor unions should actively consider the needs and views of young employees when promoting training programs, and provide effective communication, flexible training formats and visible recognition mechanisms for results. Through such efforts, the degree of resistance of young employees to training can be reduced and their career development and social contribution can be better promoted.

The government can achieve the highest economic benefits under the training mode, because it provides effective training and education at a lower cost, enabling people to acquire better skills and knowledge, thereby improving employment opportunities and economic output (46). The second is the provision of security mode, which may refer to the government to provide social welfare and security measures for young employees, such as social insurance, medical security and so on. Such a mode may be more expensive than the psychological screening mode, but it can improve the living conditions of young employees by reducing poverty and providing basic protection, thereby boosting economic growth. Finally, there is the psychological screening mode, which may refer to the government conducting psychological assessments and screenings to help people improve their mental health. Although this mode may have a positive impact on some people, compared with the first two modes, it only has a certain probability of screening out young employees with psychological problems. At the same time, this mode may require higher costs and its economic benefits may be lower.

Labor unions may focus more on ensuring workers’ interests and welfare through psychological screening and providing guarantees. This is different from coercive stress, even though coercive stress regulates job security, coordination, and psychological safety along with employee performance (4). Through psychological screening, labor unions can determine the level of depression and anxiety in young employees (47), thereby helping workers to get a job that is suitable for them. This will reduce the likelihood of psychological stress and maladjustment. In the provision of security mode, labor unions can fight for and ensure that workers receive reasonable wages, benefits and working conditions, thereby improving their overall income and living standards. In contrast, under the enhanced training mode, although workers may acquire better skills and knowledge, if the union has a low reputation, it may not be effective in negotiating for better wages and treatment. Therefore, in this case, the psychological screening and provision of guarantees mode may be more beneficial to the equilibrium gains of labor unions and workers.

Under the providing social security mode, if the reputation of the union is high, workers can rely more confidently on the union to negotiate better wages, benefits, and working conditions. Labor unions can reach more favorable labor contracts with employers through negotiation and mediation, so that workers can benefit from obtaining stable income and social security. Under the enhanced training mode, although workers can acquire better skills and knowledge through training, if the reputation of the union is high, the union can strive for better opportunities and treatment for workers. Labor unions can be involved in setting industry standards and norms that push employers to offer more equitable and competitive pay and promotion opportunities, thereby improving workers’ earnings and career advancement. Under the psychological screening mode, if the labor union has a low reputation, it may not be able to effectively fight for better treatment and rights, resulting in lower equilibrium benefits for workers.

## 5 Conclusion

The scope of each mode of improving mental health is an important question. The purpose of this study is to compare and analyze the scope of application of different modes, so as to provide reference for the government and labor unions to protect the mental health of young employees. Therefore, this article establishes three differential game models of psychological screening, providing security and strengthening training, and makes a comparative analysis of them. The main contribution of this article is to provide reference for how the government and labor unions can improve the mental health level of young employees. The research shows that under the condition that the cost paid by the government is low, the training mode can make the government obtain the maximum economic benefits. When the labor unions’ reputation improved through efforts is low, the equilibrium return of the labor union under psychological screening and providing security is the same, but greater than that under intensive training. If the union has promoted a high reputation through efforts, the guarantee mode can make the labor union obtain the highest equilibrium benefit.

This article has some shortcomings. First, the research scope of this article is global employees, not employees in a particular country. However, the role and intensity of the role played by labor unions in different countries may not be completely consistent. Second, this article is based on the game theory model to study how to improve the mental health level of young employees, and there are few specific relevant data. At the same time, this study does not involve young people in specific regions and companies, but the specific conditions faced by young employees in different countries or companies may not be exactly the same. In future studies, we can add relevant specific data to study the improvement of the mental health level of young employees in specific countries. The research of this article has certain expansibility. For example, this article argues that the protection of the mental health of young employees by the government and labor union is in line with economic principles, labor unions are concerned about their own reputation, and the mental health status of young employees is in constant change. In future studies, we can consider that the mental health of young employees conforms to other non-economic principles, that labor unions pay more attention to their own economic interests, and that the mental health status of young employees remains unchanged. Meanwhile, some gaps in the research can be solved in future research. Firstly, it is necessary to determine the specific standards for the mental health guarantee mode for young employees in different situations. Secondly, the achievements of the government and the labor unions on the mental health guarantee mode for young employees can be transformed into practical policy recommendations. Thirdly, in the process of formulating the mental health guarantee mode for young employees in different regions, the government and the labor union should determine the order of action of relevant research, rather than taking action simultaneously. In addition, this study is not only applicable to the mental health problems of young employees, but also has certain reference significance for the research on the mental health problems of left-behind children and older adults living alone.

## Data availability statement

The original contributions presented in the study are included in the article/Supplementary material, further inquiries can be directed to the corresponding author.

## Author contributions

YBa: Conceptualization, Data curation, Investigation, Methodology, Writing – original draft, Writing – review & editing. LW: Investigation, Software, Writing – original draft. YBi: Writing – original draft.
