# Supplementary material for: Psychological screening, providing social security or strengthening training? How can government and labor unions protect the mental health of young employees
Source: Front Public Health. 2023 Dec 4;11:1261286. doi: 10.3389/fpubh.2023.1261286 (PMC10726034; doi:10.3389/fpubh.2023.1261286)
Supplement: Supplementary file 1 [file Data_Sheet_1.docx]

Appendix 1

Figure 1. Relationship between three modes for improving mental health

Appendix 2

Take the derivatives of *F_S_*_1_ with respect to (13), and take the derivatives of *F_S_*_2_ with respect to (14), and set them equal to zero, we can get:

$F_{S1}^{\text{*}}\left(t\right)=\frac{a_{S1}p_{1}+\frac{\partial V_{S1}}{\partial x_{S1}}\left(p_{1}\beta_{1}-f_{1}\right)}{c_{S1}\ln\left(e+I_{S}\right)}$ (43)

$F_{S2}^{\text{*}}\left(t\right)=\frac{a_{S2}p_{2}+\frac{\partial V_{S2}}{\partial x_{S2}}\left(p_{2}\beta_{2}-f_{2}\right)}{c_{S2}}$ (44)

Substituting (43) into (13) and substituting (44) into (14), we can get: $\begin{matrix} \rho V_{S1}=a_{S1}\frac{a_{S1}p_{1}+\frac{\partial V_{S1}}{\partial x_{S1}}\left(p_{1}\beta_{1}-f_{1}\right)}{c_{S1}\ln\left(e+I_{S}\right)}p_{1}-\frac{c_{S1}\ln\left(e+I_{S}\right)}{2}\left[\frac{a_{S1}p_{1}+\frac{\partial V_{S1}}{\partial x_{S1}}\left(p_{1}\beta_{1}-f_{1}\right)}{c_{S1}\ln\left(e+I_{S}\right)}\right]^{2} \\ +lx_{S1}\left(t\right)+\frac{\partial V_{S1}}{\partial x_{S1}}\left(p_{1}\beta_{1}-f_{1}\right)\frac{a_{S1}p_{1}+\frac{\partial V_{S1}}{\partial x_{S1}}\left(p_{1}\beta_{1}-f_{1}\right)}{c_{S1}\ln\left(e+I_{S}\right)}-\frac{\partial V_{S1}}{\partial x_{S1}}\delta x_{S1}\left(t\right) \end{matrix}$ (45)

$\begin{matrix} \rho V_{S2}=a_{S2}\frac{a_{S2}p_{2}+\frac{\partial V_{S2}}{\partial x_{S2}}\left(p_{2}\beta_{2}-f_{2}\right)}{c_{S2}}p_{2}-\frac{c_{S2}}{2}\left[\frac{a_{S2}p_{2}+\frac{\partial V_{S2}}{\partial x_{S2}}\left(p_{2}\beta_{2}-f_{2}\right)}{c_{S2}}\right]^{2} \\ +lx_{S2}\left(t\right)+\frac{\partial V_{S2}}{\partial x_{S2}}\left(p_{2}\beta_{2}-f_{2}\right)\frac{a_{S2}p_{2}+\frac{\partial V_{S2}}{\partial x_{S2}}\left(p_{2}\beta_{2}-f_{2}\right)}{c_{S2}}-\frac{\partial V_{S2}}{\partial x_{S2}}\delta x_{S2}\left(t\right) \end{matrix}$ (46)

Let $V_{S1}^{*}=k_{1}x_{S1}+k_{2}$,$V_{S2}^{\text{*}}=k_{3}x_{S2}+k_{4}$, wherein, *k*_1_, *k*_2_, *k*_3_ and *k*_4_ are all constants. The parameters of the optimal social welfare function can be obtained by calculation as follows:

$\left\{\begin{matrix} k_{1}=\frac{l}{\rho+\delta} \\ k_{2}=\frac{1}{\rho}a_{S1}\frac{a_{S1}p_{1}+\frac{l}{\rho+\delta}\left(p_{1}\beta_{1}-f_{1}\right)}{c_{S1}\ln\left(e+I_{S}\right)}p_{1}-\frac{1}{\rho}\frac{c_{S1}\ln\left(e+I_{S}\right)}{2}\left[\frac{a_{S1}p_{1}+\frac{l}{\rho+\delta}\left(p_{1}\beta_{1}-f_{1}\right)}{c_{S1}\ln\left(e+I_{S}\right)}\right]^{2} \\ +\frac{1}{\rho}\frac{l}{\rho+\delta}\left(p_{1}\beta_{1}-f_{1}\right)\frac{a_{S1}p_{1}+\frac{l}{\rho+\delta}\left(p_{1}\beta_{1}-f_{1}\right)}{c_{S1}\ln\left(e+I_{S}\right)} \end{matrix}\right.$ (47)

$\left\{\begin{matrix} k_{3}=\frac{l}{\rho+\delta} \\ k_{4}=\frac{1}{\rho}a_{S2}\frac{a_{S2}p_{2}+\frac{l}{\rho+\delta}\left(p_{2}\beta_{2}-f_{2}\right)}{c_{S2}}p_{2}-\frac{1}{\rho}\frac{c_{S2}}{2}\left[\frac{a_{S2}p_{2}+\frac{l}{\rho+\delta}\left(p_{2}\beta_{2}-f_{2}\right)}{c_{S2}}\right]^{2} \\ +\frac{1}{\rho}\frac{l}{\rho+\delta}\left(p_{2}\beta_{2}-f_{2}\right)\frac{a_{S2}p_{2}+\frac{l}{\rho+\delta}\left(p_{2}\beta_{2}-f_{2}\right)}{c_{S2}} \end{matrix}\right.$ (48)

Therefore, it can be concluded that:

$\begin{matrix} V_{S1}^{*}=\frac{l}{\rho+\delta}x_{S1}+\frac{1}{\rho}a_{S1}\frac{a_{S1}p_{1}+\frac{l}{\rho+\delta}\left(p_{1}\beta_{1}-f_{1}\right)}{c_{S1}\ln\left(e+I_{S}\right)}p_{1}-\frac{1}{\rho}\frac{c_{S1}\ln\left(e+I_{S}\right)}{2}\left[\frac{a_{S1}p_{1}+\frac{l}{\rho+\delta}\left(p_{1}\beta_{1}-f_{1}\right)}{c_{S1}\ln\left(e+I_{S}\right)}\right]^{2} \\ +\frac{1}{\rho}\frac{l}{\rho+\delta}\left(p_{1}\beta_{1}-f_{1}\right)\frac{a_{S1}p_{1}+\frac{l}{\rho+\delta}\left(p_{1}\beta_{1}-f_{1}\right)}{c_{S1}\ln\left(e+I_{S}\right)} \end{matrix}$ (49)

$\begin{matrix} V_{S2}^{\text{*}}=\frac{l}{\rho+\delta}x_{S2}+\frac{1}{\rho}a_{S2}\frac{a_{S2}p_{2}+\frac{l}{\rho+\delta}\left(p_{2}\beta_{2}-f_{2}\right)}{c_{S2}}p_{2}-\frac{1}{\rho}\frac{c_{S2}}{2}\left[\frac{a_{S2}p_{2}+\frac{l}{\rho+\delta}\left(p_{2}\beta_{2}-f_{2}\right)}{c_{S2}}\right]^{2} \\ +\frac{1}{\rho}\frac{l}{\rho+\delta}\left(p_{2}\beta_{2}-f_{2}\right)\frac{a_{S2}p_{2}+\frac{l}{\rho+\delta}\left(p_{2}\beta_{2}-f_{2}\right)}{c_{S2}} \end{matrix}$ (50)

In this case,

$F_{S1}^{\text{*}}\left(t\right)=\frac{a_{S1}p_{1}+\frac{l}{\rho+\delta}\left(p_{1}\beta_{1}-f_{1}\right)}{c_{S1}\ln\left(e+I_{S}\right)}$ (51)

$F_{S2}^{\text{*}}\left(t\right)=\frac{a_{S2}p_{2}+\frac{l}{\rho+\delta}\left(p_{2}\beta_{2}-f_{2}\right)}{c_{S2}}$ (52)

Appendix 3

Take the derivatives of *F_G_*_1_ with respect to (15), and take the derivatives of *F_G_*_2_ with respect to (16), and set them equal to zero, we can get:

$F_{G1}^{\text{*}}\left(t\right)=\frac{a_{G1}+\frac{\partial V_{G1}}{\partial x_{G1}}\beta_{1}}{c_{G1}}$ (53)

$F_{G2}^{\text{*}}\left(t\right)=\frac{\left(a_{G2}-b_{G2}\right)+\frac{\partial V_{G2}}{\partial x_{G2}}\beta_{2}}{c_{G2}}$ (54)

Substituting (53) into (15) and substituting (54) into (16), we can get:

$\rho V_{G1}=a_{G1}\frac{a_{G1}+\frac{\partial V_{G1}}{\partial x_{G1}}\beta_{1}}{c_{G1}}-\frac{c_{G1}}{2}\left[\frac{a_{G1}+\frac{\partial V_{G1}}{\partial x_{G1}}\beta_{1}}{c_{G1}}\right]^{2}+lx_{G1}\left(t\right)+\frac{\partial V_{G1}}{\partial x_{G1}}\beta_{1}\frac{a_{G1}+\frac{\partial V_{G1}}{\partial x_{G1}}\beta_{1}}{c_{G1}}-\frac{\partial V_{G1}}{\partial x_{G1}}\delta x_{G1}\left(t\right)$(55)

$\begin{matrix} \rho V_{G2}=a_{G2}\frac{\left(a_{G2}-b_{G2}\right)+\frac{\partial V_{G2}}{\partial x_{G2}}\beta_{2}}{c_{G2}}-\frac{c_{G2}}{2}\left[\frac{\left(a_{G2}-b_{G2}\right)+\frac{\partial V_{G2}}{\partial x_{G2}}\beta_{2}}{c_{G2}}\right]^{2}-b_{G2}\frac{\left(a_{G2}-b_{G2}\right)+\frac{\partial V_{G2}}{\partial x_{G2}}\beta_{2}}{c_{G2}} \\ +lx_{G2}\left(t\right)+\frac{\partial V_{G2}}{\partial x_{G2}}\beta_{2}\frac{\left(a_{G2}-b_{G2}\right)+\frac{\partial V_{G2}}{\partial x_{G2}}\beta_{2}}{c_{G2}}-\frac{\partial V_{G2}}{\partial x_{G2}}\delta x_{G2}\left(t\right) \end{matrix}$ (56)

Let $V_{G1}^{*}=k_{5}x_{G1}+k_{6}$,$V_{G2}^{\text{*}}=k_{7}x_{G2}+k_{8}$, wherein, *k*_5_, *k*_6_, *k*_7_ and *k*_8_ are all constants. The parameters of the optimal social welfare function can be obtained by calculation as follows:

$\left\{\begin{matrix} k_{5}=\frac{l}{\rho+\delta} \\ k_{6}=\frac{1}{\rho}a_{G1}\frac{a_{G1}+\frac{l}{\rho+\delta}\beta_{1}}{c_{G1}}-\frac{1}{\rho}\frac{c_{G1}}{2}\left[\frac{a_{G1}+\frac{l}{\rho+\delta}\beta_{1}}{c_{G1}}\right]^{2}+\frac{1}{\rho}\frac{l}{\rho+\delta}\beta_{1}\frac{a_{G1}+\frac{l}{\rho+\delta}\beta_{1}}{c_{G1}} \end{matrix}\right.$ (57)

$\left\{\begin{matrix} k_{7}=\frac{l}{\rho+\delta} \\ k_{8}=\frac{1}{\rho}a_{G2}\frac{\left(a_{G2}-b_{G2}\right)+\frac{l}{\rho+\delta}\beta_{2}}{c_{G2}}-\frac{c_{G2}}{2}\frac{1}{\rho}\left[\frac{\left(a_{G2}-b_{G2}\right)+\frac{l}{\rho+\delta}\beta_{2}}{c_{G2}}\right]^{2}-\frac{1}{\rho}b_{G2}\frac{\left(a_{G2}-b_{G2}\right)+\frac{l}{\rho+\delta}\beta_{2}}{c_{G2}} \\ +\frac{1}{\rho}\frac{l}{\rho+\delta}\beta_{2}\frac{\left(a_{G2}-b_{G2}\right)+\frac{l}{\rho+\delta}\beta_{2}}{c_{G2}} \end{matrix}\right.$(58)

Therefore, it can be concluded that:

$V_{G1}^{*}=\frac{l}{\rho+\delta}x_{G1}+\frac{1}{\rho}a_{G1}\frac{a_{G1}+\frac{l}{\rho+\delta}\beta_{1}}{c_{G1}}-\frac{1}{\rho}\frac{c_{G1}}{2}\left[\frac{a_{G1}+\frac{l}{\rho+\delta}\beta_{1}}{c_{G1}}\right]^{2}+\frac{1}{\rho}\frac{l}{\rho+\delta}\beta_{1}\frac{a_{G1}+\frac{l}{\rho+\delta}\beta_{1}}{c_{G1}}$ (59)

$\begin{matrix} V_{G2}^{\text{*}}=\frac{l}{\rho+\delta}x_{G2}+\frac{1}{\rho}a_{G2}\frac{\left(a_{G2}-b_{G2}\right)+\frac{l}{\rho+\delta}\beta_{2}}{c_{G2}}-\frac{c_{G2}}{2}\frac{1}{\rho}\left[\frac{\left(a_{G2}-b_{G2}\right)+\frac{l}{\rho+\delta}\beta_{2}}{c_{G2}}\right]^{2} \\ -\frac{1}{\rho}b_{G2}\frac{\left(a_{G2}-b_{G2}\right)+\frac{l}{\rho+\delta}\beta_{2}}{c_{G2}}+\frac{1}{\rho}\frac{l}{\rho+\delta}\beta_{2}\frac{\left(a_{G2}-b_{G2}\right)+\frac{l}{\rho+\delta}\beta_{2}}{c_{G2}} \end{matrix}$ (60)

In this case,

$F_{G1}^{\text{*}}\left(t\right)=\frac{a_{G1}+\frac{l}{\rho+\delta}\beta_{1}}{c_{G1}}$ (61)

$F_{G2}^{\text{*}}\left(t\right)=\frac{\left(a_{G2}-b_{G2}\right)+\frac{l}{\rho+\delta}\beta_{2}}{c_{G2}}$ (62)

Appendix 4

Take the derivatives of *F_T_*_1_ with respect to (17), and take the derivatives of *F_T_*_2_ with respect to (18), and set them equal to zero, we can get:

$F_{T1}^{\text{*}}\left(t\right)=\frac{\left(a_{T1}+b_{T1}\right)+\frac{\partial V_{T1}}{\partial x_{T1}}\left(\beta_{1}-d\right)}{c_{T1}}$ (63)

$F_{T2}^{\text{*}}\left(t\right)=\frac{a_{T2}+\frac{\partial V_{T2}}{\partial x_{T2}}\left(\beta_{2}-d\right)}{c_{T2}}$ (64)

$\begin{matrix} \rho V_{T1}=a_{T1}\frac{\left(a_{T1}+b_{T1}\right)+\frac{\partial V_{T1}}{\partial x_{T1}}\left(\beta_{1}-d\right)}{c_{T1}}-\frac{c_{T1}}{2}\left[\frac{\left(a_{T1}+b_{T1}\right)+\frac{\partial V_{T1}}{\partial x_{T1}}\left(\beta_{1}-d\right)}{c_{T1}}\right]^{2}+\frac{\left(a_{T1}+b_{T1}\right)+\frac{\partial V_{T1}}{\partial x_{T1}}\left(\beta_{1}-d\right)}{c_{T1}} \\ \cdot b_{T1}+lx_{T1}\left(t\right)+\frac{\partial V_{T1}}{\partial x_{T1}}\left(\beta_{1}-d\right)\frac{\left(a_{T1}+b_{T1}\right)+\frac{\partial V_{T1}}{\partial x_{T1}}\left(\beta_{1}-d\right)}{c_{T1}}-\frac{\partial V_{T1}}{\partial x_{T1}}\delta x_{T1}\left(t\right) \end{matrix}$(65)$\begin{matrix} \rho V_{T2}=a_{T2}\frac{a_{T2}+\frac{\partial V_{T2}}{\partial x_{T2}}\left(\beta_{2}-d\right)}{c_{T2}}-\frac{c_{T2}}{2}\left[\frac{a_{T2}+\frac{\partial V_{T2}}{\partial x_{T2}}\left(\beta_{2}-d\right)}{c_{T2}}\right]^{2}+lx_{T2}\left(t\right) \\ +\frac{\partial V_{T2}}{\partial x_{T2}}\left(\beta_{2}-d\right)\frac{a_{T2}+\frac{\partial V_{T2}}{\partial x_{T2}}\left(\beta_{2}-d\right)}{c_{T2}}-\frac{\partial V_{T2}}{\partial x_{T2}}\delta x_{T2}\left(t\right) \end{matrix}$ (66)

Let $V_{T1}^{*}=k_{9}x_{T1}+k_{10}$,$V_{G2}^{\text{*}}=k_{11}x_{G2}+k_{12}$, wherein, *k*_9_, *k*_10_, *k*_11_ and *k*_12_ are all constants. The parameters of the optimal social welfare function can be obtained by calculation as follows:

$\left\{\begin{matrix} k_{9}=\frac{l}{\rho+\delta} \\ k_{10}=\frac{1}{\rho}a_{T1}\frac{\left(a_{T1}+b_{T1}\right)+\frac{l}{\rho+\delta}\left(\beta_{1}-d\right)}{c_{T1}}-\frac{1}{\rho}\frac{c_{T1}}{2}\left[\frac{\left(a_{T1}+b_{T1}\right)+\frac{l}{\rho+\delta}\left(\beta_{1}-d\right)}{c_{T1}}\right]^{2} \\ +\frac{1}{\rho}b_{T1}\frac{\left(a_{T1}+b_{T1}\right)+\frac{l}{\rho+\delta}\left(\beta_{1}-d\right)}{c_{T1}}+\frac{1}{\rho}\frac{l}{\rho+\delta}\left(\beta_{1}-d\right)\frac{\left(a_{T1}+b_{T1}\right)+\frac{l}{\rho+\delta}\left(\beta_{1}-d\right)}{c_{T1}} \\ \end{matrix}\right.$ (67)

$\left\{\begin{matrix} k_{11}=\frac{l}{\rho+\delta} \\ k_{12}=\frac{1}{\rho}a_{T2}\frac{a_{T2}+\frac{l}{\rho+\delta}\left(\beta_{2}-d\right)}{c_{T2}}-\frac{1}{\rho}\frac{c_{T2}}{2}\left[\frac{a_{T2}+\frac{l}{\rho+\delta}\left(\beta_{2}-d\right)}{c_{T2}}\right]^{2} \\ +\frac{l}{\rho+\delta}\left(\beta_{2}-d\right)\frac{a_{T2}+\frac{l}{\rho+\delta}\left(\beta_{2}-d\right)}{c_{T2}} \\ \end{matrix}\right.$ (68)

Therefore, it can be concluded that:

$\begin{matrix} V_{T1}^{*}=\frac{l}{\rho+\delta}x_{T1}+\frac{1}{\rho}a_{T1}\frac{\left(a_{T1}+b_{T1}\right)+\frac{l}{\rho+\delta}\left(\beta_{1}-d\right)}{c_{T1}}-\frac{1}{\rho}\frac{c_{T1}}{2}\left[\frac{\left(a_{T1}+b_{T1}\right)+\frac{l}{\rho+\delta}\left(\beta_{1}-d\right)}{c_{T1}}\right]^{2} \\ +\frac{1}{\rho}b_{T1}\frac{\left(a_{T1}+b_{T1}\right)+\frac{l}{\rho+\delta}\left(\beta_{1}-d\right)}{c_{T1}}+\frac{1}{\rho}\frac{l}{\rho+\delta}\left(\beta_{1}-d\right)\frac{\left(a_{T1}+b_{T1}\right)+\frac{l}{\rho+\delta}\left(\beta_{1}-d\right)}{c_{T1}} \end{matrix}$ (69)

$\begin{matrix} V_{T2}^{\text{*}}=\frac{l}{\rho+\delta}x_{T2}+\frac{1}{\rho}a_{T2}\frac{a_{T2}+\frac{l}{\rho+\delta}\left(\beta_{2}-d\right)}{c_{T2}}-\frac{1}{\rho}\frac{c_{T2}}{2}\left[\frac{a_{T2}+\frac{l}{\rho+\delta}\left(\beta_{2}-d\right)}{c_{T2}}\right]^{2} \\ +\frac{l}{\rho+\delta}\left(\beta_{2}-d\right)\frac{a_{T2}+\frac{l}{\rho+\delta}\left(\beta_{2}-d\right)}{c_{T2}} \end{matrix}$ (70)

In this case,

$F_{T1}^{\text{*}}\left(t\right)=\frac{\left(a_{T1}+b_{T1}\right)+\frac{l}{\rho+\delta}\left(\beta_{1}-d\right)}{c_{T1}}$ (71)

$F_{T2}^{\text{*}}\left(t\right)=\frac{a_{T2}+\frac{l}{\rho+\delta}\left(\beta_{2}-d\right)}{c_{T2}}$ (72)
